# Warriors of Science: Ukraine’s Academic Endeavors

**DOI:** 10.1117/1.NPh.10.3.030101

**Published:** 2023-08-24

**Authors:** Daria Bogatova

**Affiliations:** Boston University, College of Arts and Sciences, Department of Biology, Boston, Massachusetts, United States

## Abstract

A special editorial considers the narratives of four scientists from Ukraine.

“We were told: you have no other option but to surrender.

We say: we have no other option than to win.”

- Volodymyr Zelensky

In a world where science is often relegated to sterile laboratories, intricate textbooks, and cerebral theories, we risk forgetting the human courage that fuels its progress — the valor of researchers, the heroism of scientists, the dauntless minds ceaselessly pushing the boundaries of our understanding. Today, as Ukraine stands resilient on its 32^nd^ Independence Day, bearing the scars but also the strength gained from a full-scale invasion just a year and a half ago, we illuminate the tales of grit and heroism that shape its scientific community.

In this special editorial, we venture into the stirring narratives of four scientists. They hail from various corners of Ukraine, yet they share a common thread: an unwavering dedication to their scientific endeavors, standing resilient amidst the turbulence of war.

## Visions of Calm

**Figure:**
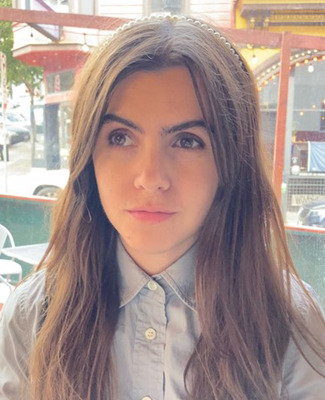


**Solomia Savchuk** was born in Kolomyya and is pursuing an MD/MS degree at Stanford University. She is at the forefront of the revolutionary field of cancer neuroscience, working on understanding how neurons interact with the microenvironment of small-cell lung cancer, thus promoting or hindering its growth. Before the full-scale invasion, her life revolved around rigorous medical studies and research. Today, she stands tall, helming an impressive force of over 200 volunteers, tirelessly helping those in need.

**Figure:**
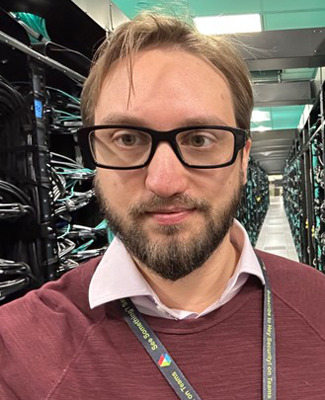


**Oleksandr Narykov** comes from Zaporizhzhia, a city close to the nuclear power plant currently occupied. By diving into alternative splicing and its influence on neural functions, Oleksandr hopes to offer therapeutic solutions for neurodegenerative diseases as well as to contribute to fundamentals of neuroscience. At the Argonne National Laboratory, his work has the potential to change our understanding of neural cells and the brain’s intricacies. But his love for science is paralleled by his dedication to Ukraine. Despite the war’s emotional weight, he has engaged in humanitarian efforts, proving that his spirit, much like his scientific curiosity, remains unyielding.

**Figure:**
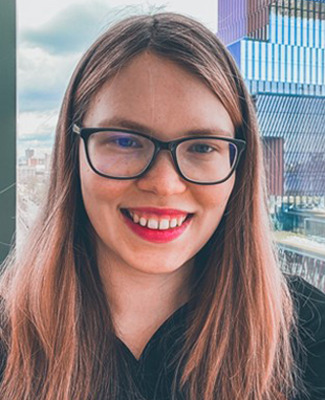


**Anna Novoseltseva** currently working on her PhD degree in Biomedical Engineering at Boston University, was born in Ulan-Ude in Russia. With a laser-focus on optical tools, her mission is to unravel the mysteries of myelin changes and shed light on conditions like Alzheimer’s and chronic traumatic encephalopathy through polarized light studies. Anna’s story is unique since she spent over a year in Ukraine during the COVID-19 pandemic.

**Figure:**
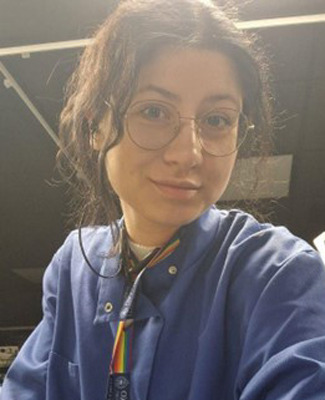


**Olena Didenko** was born in the city of Mykolaiv. She is an alumna of Taras Shevchenko University, Ukraine’s premier institution, where she earned her bachelor’s degree in biology. Now on the cusp of completing her master’s degree in neuroscience at the University of Oxford, Olena’s groundbreaking work in behavioral neuroscience delves into visual decision-making in mice. Her research holds promising potential for advancing treatments in various neurological conditions.

## Onset of Chaos

February 24th 2022.

Thursday.

4 am.

Air Raid.

Big red letters saying “PUTIN DECLARED WAR.”

Ukrainians all over the world awoke with souls forever altered.

**Solomia:**

“I was prepping for a lab meeting, simultaneously juggling manual tasks and compulsively updating my browser tabs. Honestly, I was in denial; the idea of war seemed inconceivable in today’s age.”

The magnitude of the catastrophe weighed heavily on Solomia. Her friends, many of whom resided in Ivano-Frankivsk and Kyiv regions, were now in the direct line of fire, with nearby airports coming under shell attacks. Panic gripped her as she thought of the safety of those close to her, especially when considering the daunting task of evacuating with a small child in tow. Over the next 24 hours, she found herself glued to the screen, watching her homeland grapple with a harrowing assault. Amid this chaos, there was a silver lining – an outpouring of support came her way. Emails flooded her inbox from faculty, advisors, and peers. Each message, though filled with genuine concern and warmth, became increasingly challenging for Solomia to respond to. The gravity of the situation created a chasm between her current reality and the world she once knew at Stanford.

**Anna:**

“The moment I learned about the war, I felt an overwhelming shock. My fingers frantically dialed numbers, reaching out to friends in Ukraine, praying they were safe. They were not.”

Tears flooded her eyes as she grappled with the unfolding tragedy. “It’s heart-wrenching,” she said. “Ukrainians are some of the kindest souls I’ve met, and it’s just cruel what our government is doing.”

Her time living in Ukraine painted a picture far removed from the current chaos. The delightful flavors of the food, the generosity of people; everything was a testament to the warmth of the Ukrainian spirit. Whenever there was a language barrier, locals would seamlessly switch between Russian and English to ensure Anna felt welcomed. This genuine kindness stood in stark contrast to the image portrayed by Russian propaganda.

But now, a cloud of fear hangs over Anna’s longing to return to her homeland. The war has not only distanced her from her beloved Ukraine but also created rifts within her family due to differing viewpoints. The conflict has deepened Anna’s spirituality, prompting her to pray more fervently, not just for peace but also for enlightenment for the Russian people.

**Oleksandr:**

“In Boston, surrounded by my closest friends, I found myself frozen in front of the screen as Putin delivered his speech. The news was almost surreal until my friends began receiving updates about explosions in Kyiv. Without hesitation, I dialed home, rousing my parents from their sleep. At first, their voices held a calmness, perhaps a touch of disbelief. But within minutes, the distant roar of explosions punctuated our conversation. The Russians had targeted an airport in Zaporizhzhya. The reality of the situation hit hard, and the distance from home never felt more profound.”

**Olena:**

Olena was at home in Kyiv when the war started.

“We woke up at around 5 am when our relatives tried to call and tell us that the war had begun. The specifics of the conversation may elude me, but the overwhelming waves of fear and frustration I felt are etched indelibly in my memory. Looking out the window, the streets were chaotic. People were rushing about, cars honked incessantly, and everywhere there was a sense of panic. My emergency backpack, prepared and waiting for weeks, stood ready in the hall. Yet, in that moment, I was overwhelmed, unsure of our next steps in the unfolding chaos. All I wanted was to feel safe. It was the scariest thing I had ever experienced, and the events left a lasting impact on me, shaping how I see things both as a researcher and as a person.”

## Life Altered

In the wake of those paralyzing first hours, a stark clarity emerged. Ukrainians, scattered worldwide, ignited a global call to arms. Some brandished weapons at the frontlines, others breathed life back in hospital wards, drove trucks brimming with aid, or stood defiantly at rallies amplifying their voice. Every soul had a purpose, a mission. Everyone became a warrior.

**Oleksandr and Olena:**

In the face of Ukraine’s upheaval, both Oleksandr and Olena stepped forward, driven by a shared devotion to their homeland. Oleksandr swiftly joined volunteer organizations, aiding refugees and the armed forces, he now actively supports Ukraine’s armed forces and rehabilitates soldiers from Chicago. On the other hand, Olena, deeply embedded in her local Ukrainian community, organizes fundraisers and champions peaceful demonstrations. She emphasizes the collective might of individual donations, asserting that even small contributions, akin to the cost of a coffee, can resonate powerfully when pooled together.

**Anna:**

Anna’s calling was to help displaced families. Encountering a family of refugees with two young children, the weight of their distress and vulnerability was palpable to her. Anna took an extraordinary step. She gave them every penny from her bank account, a decision driven by an overpowering need to shield them, especially the innocent children, from the harsh reality of being stranded on the streets. In the midst of overwhelming adversity, Anna’s act of kindness became a vivid embodiment of human empathy and boundless altruism.

**Solomia:**

Prior to the war, Solomia had little engagement with the Ukrainian community. However, the very next day saw the beginnings of organized demonstrations. Without an official student body or even a budget to support their cause, she and her peers would huddle together post school hours, dedicating 5-6 hours daily, passionately working on a slew of projects. This wasn’t just a side hustle; it became a pressing obligation alongside their regular academic commitments.

“Telehelp Ukraine emerged from this crucible of determination. What started as a humble student initiative at Stanford transformed into a formidable force. With over 200 volunteers, we’ve already seen more than 2000 patients, some even from within the heart of occupied regions. Physicians, therapists, mental health experts — we’ve all banded together, bridging the gap for those cut off from essential healthcare during this crisis.

The trauma we witness is staggering, its depth unfathomable. But we’re not ones to back down. In places where communication seems impossible, where villages on the frontline without internet connection or even electricity, we rise to the challenge. Our volunteers personally drive down, set up Starlink connections, ensuring that every individual gets the care they desperately need.”

## Innovating for the Future

**Solomia:**

Balancing between the rigorous demands of medicine, the drive to support her homeland, and personal growth hasn’t been straightforward for Solomia. “It’s an evolving process,” she admits, tackling challenges one day at a time. While she leads a substantial team, she wrestles with feelings of not doing enough, both for Ukraine and in her professional sphere.

In envisioning her future, Solomia is steadfast in her commitment to medical science. She plans to complete her medical studies and delve deeper into the realm of science, striving to strike the right balance between research and hands-on clinical practice. But her aspirations don’t end at only self-achievement. Solomia hopes to share her expertise with both her homeland and the international community. “I dream of practicing medicine and teaching in Ukraine,” she confides, emphasizing her desire to maintain ties with the U.S. institutions that nurtured her capabilities. She acknowledges the massive reconstruction needed in Ukraine’s medical facilities and is determined to channel her skills where they’re most crucial.

**Anna:**

In her professional journey Anna is determined to conquer the frontiers of neuroscience, ardently seeking cures for Alzheimer’s disease and chronic traumatic encephalopathy. With a heavy heart, she wishes Victory for her Ukrainian colleagues and friends.

“Stay strong and never surrender. I envision a day when we all return home to rejoice in our shared triumph.”

**Oleksandr:**

“My mission is clear: I want to utilize my computer science background to create smarter AI, aiming to revolutionize healthcare for everyone.

The urgency to improve healthcare isn’t just academic; it’s deeply personal. For me, pushing the boundaries in healthcare is more than just professional growth; it’s my way of giving back to a global community that stood with Ukraine during its most challenging times. I’m profoundly thankful for the unwavering support from the U.S. and my collaborating institutions, as together, we continue to push the boundaries of science.”

**Olena:**

“In a world where cruelty exists, we mustn’t let it change our core. Every day, I stand up for what’s dear to me: my homeland, my family, the people I love, and the values I cherish. These aren’t just feelings; they fuel my purpose and drive.

I might not have the power to change the world, but I have a unique gift – my expertise in neuroscience. I can make a real difference for those battling neurological challenges. It’s deeply saddening to see people suffer through no fault of their own, especially when faced with health challenges from birth.

But here’s the thing: if my knowledge can bring a ray of hope or ease someone’s pain, even just a bit, then I’ve made a difference. Every small act, every moment I can bring relief to someone, adds meaning to my life. If I can positively touch even one life, it’s all worth it.”

In the crucible of war, these four young scientists have been tempered, not weakened. The devastation of the fights they endured in Ukraine resonates with a universal struggle – the battles that countless individuals face against relentless diseases. These battles, just like the war, often come unchosen, unexpected, and full of anguish. Yet, like their homeland’s resilience, these scientists have emerged with an even fiercer determination and a profound understanding of the essence of struggle. Their firsthand experience of war has deepened their commitment to helping others confront their personal health battles. Now, they’re channeling their strength, not just to rebuild their nation, but to assist individuals worldwide in their own wars against illness. Their journey underscores a potent truth: adversity, while formidable, can also forge the most powerful agents of hope and healing.

Happy Independence Day, Ukraine!

Slava Ukraini!

